# ﻿*Oreocharisphuongii* (Gesneriaceae), a new species from central Vietnam

**DOI:** 10.3897/phytokeys.193.77083

**Published:** 2022-03-17

**Authors:** Khuong Duy Le, Thanh Trung Nguyen, Phuong Thanh Nguyen, Thao Thi Hoang, Fang Wen, Truong Van Do

**Affiliations:** 1 Centre for Research on Ha Long Bay, Faculty of Environment, Ha Long University, 258; 2 th; 3 Bach Dang, Uong Bi, Quang Ninh, Vietnam; 4 Graduate University of Science and Technology, Vietnam Academy of Science & Technology, 18; 5 th; 6 Hoang Quoc Viet Road, Cau Giay, Hanoi, Vietnam; 7 Vietnam National Museum of Nature, Vietnam Academy of Science & Technology, 18; 8 th; 9 Hoang Quoc Viet Road, Cau Giay, Hanoi, Vietnam; 10 Faculty of Biology, VNU University of Science, 334 Nguyen Trai, Thanh Xuan, Hanoi, Vietnam; 11 Bac Giang Agriculture and Forestry University, Bich Dong, Viet Yen, Bac Giang, Vietnam; 12 Guangxi Key Laboratory of Plant Conservation and Restoration Ecology in Karst Terrain, Guangxi Institute of Botany, Guangxi Zhuang Autonomous Region and Chinese Academy of Sciences, CN-541006 Guilin, China; 13 Gesneriad Committee of China Wild Plant Conservation Association, National Gesneriaceae Germplasm Resources Bank of GXIB, Gesneriad Conservation Center of China (GCCC), CN-541006 Guilin, Guangxi, China

**Keywords:** Annamite Range, Didymocarpoideae, endemic to Vietnam, Flora of Vietnam, limestone flora, new taxon

## Abstract

*Oreocharisphuongii*, a new species of Gesneriaceae from central Vietnam, is described and illustrated here. The new species is most similar to *O.longifolia* by sharing peduncles up to 22 cm long, bracts 2, zygomorphic, yellow flowers with tubular corolla, stamens 4 with two pairs of coherent anthers and capsules up to 6 cm long. It mainly differs from the latter by the combination of some morphological characters of leaves (shape, base, apex and margin), size of calyx lobes, indumentum of corolla tube and inner surface of three lower corolla lobes. Detailed morphological description together with colour illustration, information on phenology, distribution, ecology, preliminarily conservation status of the new species and comparison with its similar species are also presented.

## ﻿Introduction

The genus *Oreocharis* Bentham (Gesneriaceae DC), prior to recent phylogenetic work, comprised ca. 28 species, mainly distributed in southern China (Li and Wang 2004). Recent molecular and morphology-based analyses demonstrated that the traditionally-defined *Oreocharis* was phylogenetically intertwined with nine previously defined small genera and acaulescent, rosette forming members of *Briggsia* Craib. The re-circumscribed *Oreocharis* is a strongly supported monophyletic group and placed in the subfamily Didymocarpoideae (Möller et al. 2011; 2014; Middleton et al. 2013). Since its re-definition in 2011, *Oreocharis* s.l. comprises about 150 species making the genus one of the most morphologically diverse amongst Old World Gesneriaceae (Möller et al. 2016; Möller 2019; Jin et al. 2021). Most of the approximately 130 species are found in southern and south-western China, with a few species also in northern Vietnam, India, Bhutan, Myanmar, Thailand and Japan (e.g. Pham 2000; Vu 2005, 2018; Wei et al. 2010; Möller et al. 2011, 2016, 2018; Do et al. 2017; Chen et al. 2017, 2018; Yang et al. 2019; Cai et al. 2019; Jin et al. 2021).

Prior to this work, eight species of *Oreocharis* were known from Vietnam, of which seven new species were described from the country since 2017, i.e. *O.caobangensis* T.V.Do, Y.G.Wei & F.Wen (Do et al. 2017), *O.argyrophylla* W.H.Chen, H.Q.Nguyen & Y.M.Shui, *O.blepharophylla* W.H.Chen, H.Q.Nguyen & Y.M.Shui (Chen et al. 2017), *O.grandiflora* W.H.Chen, Q.H.Nguyen & Y.M.Shui, *O.longituba* W.H.Chen, Q.H.Nguyen & Y.M.Shui (Chen et al. 2018), *O.tribracteata* Bramley, H.J.Atkins & Mich.Möller and *O.rufescens* D.J.Middleton (Möller et al. 2018). All these species have been found in close proximity or with sympatric distributions in northern Vietnam, but none of them has been recorded from the central and southern areas of the country. Due to similarities of topography and ecological factors, the flora of northern Vietnam is similar to that of south-western and southern China, which is considered the centre of *Oreocharis* diversity (Jin et al. 2021). Additionally, there are still many parts of northern and central Vietnam (such as the Annamite Range) from which specimens of vascular plants, in general and Gesneriaceae, in particular, have not yet been well collected or for which the collecting density is very low. Hence, our understanding of the diversity and distribution of vascular plant species, in general and Gesneriaceae species, in particular, in these regions remains rudimentary.

While revising the taxonomy of Gesneriaceae for the Flora of Vietnam, we have conducted numerous field investigations throughout the country and collected some interesting Gesneriaceae specimens from two populations in some protected forest areas within the Annamite Range in central Vietnam. These specimens are characterised by leaves in a basal rosette, 2-paired stamens, ring-like disc and loculicidal capsules. Due to the above characteristics, we determined that these specimens belong to *Oreocharis* s.l. Detailed morphological comparisons with the protologues and type specimens of all previously-described species of *Oreocharis* s.l. (Pan 1987; Wang et al. 1990, 1998; Li and Wang 2004; Wei et al. 2010; Liu et al. 2012; Chen et al. 2013, 2014, 2015, 2016, 2017, 2018; Tan et al. 2013, 2015; Möller et al. 2014, 2018; Möller 2015; Li and Li 2015; Yang et al. 2015a, 2015b, 2017, 2019; Wei et al. 2016; Li et al. 2017; Do et al. 2017; Cai et al. 2019, 2020; Cai and Dao 2020) revealed that these specimens do not match with any known *Oreocharis* species. Therefore, we confirmed that these specimens represent a new species, which is here described and illustrated, namely *O.phuongii* T.V.Do.

## ﻿Taxonomic treatment

### ﻿ Oreocharis phuongii

Taxon classificationPlantaePasseriformesParamythiidae

T.V.Do sp. nov.

E4CA6C5F-BB46-50D5-A68A-E97BAD099F0F

urn:lsid:ipni.org:names:77296017-1

Figs 1 , 2 , 3

#### Type.

Vietnam. Thua Thien Hue Province, Nam Dong District, Thuong Lo Commune, Bach Ma National Park, on moist rocks under evergreen broad-leaved forests, 16°07'56.5"N 107°45'03.2"E, ca. 545 m alt., 21 Nov 2019, Do Van Truong ĐVT 368 (holotype: VNMN!; isotypes: IBK!, VNMN!).

**Figure 1.**
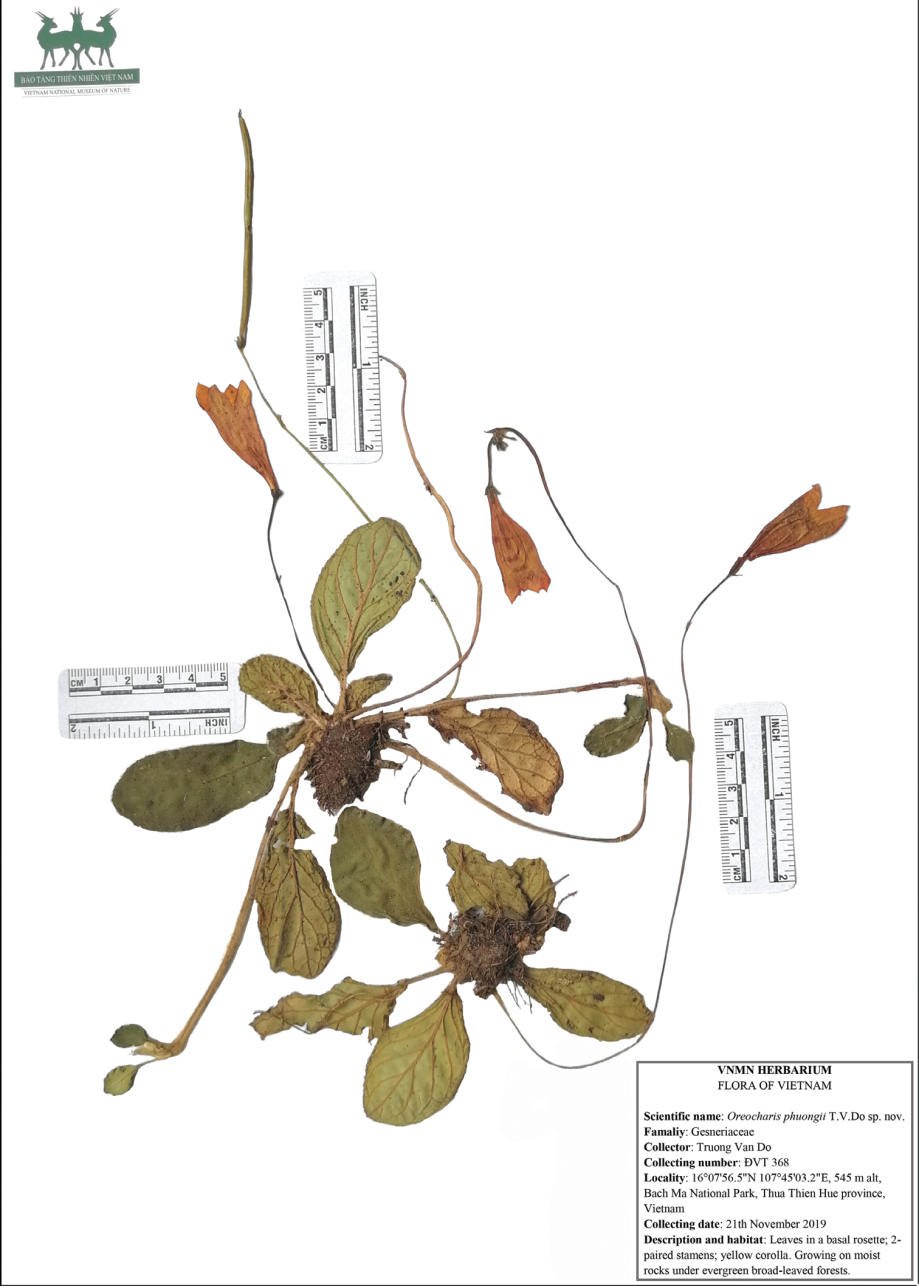
Holotype of *Oreocharisphuongii* sp. nov. (*Truong Van Do ĐVT* 368, deposited at VNMN).

#### Diagnosis.

The new species is morphologically similar to *O.longifolia* W.H.Chen in having peduncles up to 22 cm long, bracts 2, zygomorphic, yellow flowers with tubular corolla, stamens 4 and capsules up to 6 cm long; but it differs from the latter in its elliptic to ovate lamina (vs. narrowly elliptic to oblanceolate), cuneate to nearly rounded leaf base (vs. attenuate), obtuse to almost rounded leaf apex (vs. acute), crenulate margin (vs. serrulate), 3–4.5 × 1.5–1.8 mm calyx lobe size (vs. 5–7 × 2–3 mm), glabrous inner surface of corolla tube (vs. sparsely glandular puberulent with dark-purple striations) and exclusively yellow inner surface of three lower lobes without dots and striations (vs. brown to brownish-yellow with purple dots and striations).

#### Description.

Perennial acaulescent herbs, with conspicuous 12–14 cm long stolons, densely brown woolly, with (6–)8–14 leaves in a basal rosette. Petioles 1–3 cm long, densely brown villous. Leaf-blade elliptic to ovate, 4–7 × 2–2.8 cm, base cuneate to nearly rounded, apex obtuse to almost rounded, margin crenulate, adaxially dark-green, densely grey puberulous, abaxially pale-green, densely grey puberulous and browner appressed villous on main veins, secondary veins pinnate, 4–5 pairs, tertiary veins reticulate, lightly sunken on adaxial surface and conspicuously prominent on abaxial surface. Inflorescences cymose, subumbel-like, axillary, 2–3 cymes, each 1–3-flowered; peduncles 12–19(–22) cm long, erect, brown, sparsely villous to pubescent; bracts 2, linear-lanceolate to elliptic, 2–5 × 1.5–2 mm, outside with sparse, brown hairs, inside glabrescent, margin entire; pedicels 2–3 cm long, with sparse, brown hairs, sometimes bearing additional bracts at 1/2 to upper 1/3 of pedicel length, similar in size and morphology with bracts at branching points. Calyx equally 5-lobed, free to base, lobes triangular to lanceolate, 3–4.5 × 1.5–1.8 mm, both surfaces with a dense covering of long gland-tipped and eglandular hairs, margin entire. Corolla, zygomorphic, yellow, bilabiate, outside sparsely pubescent, inside glabrous; tube tubular, 28–30 × 12–13 mm, abrubtly constricted at base, 8–9 × 2.5–3 mm; upper lip slightly 2-lobed; lobes ovate, 5–7 × 4.5–5 mm, incurved backwards, apex obtuse to acute; lower lip 3-lobed, lobes elliptic, broadly ovate to semi-orbicular; lateral lobes 8–9 × 5–6 mm; middle lobe broader than lateral lobes, 8–10 × 6–7.5 mm, apex obtuse to acute or rounded. Stamens 4, anthers coherent in two pairs, filaments linear, glabrous; filaments of upper pair 15–17 mm long, adnate at 7–8 mm from the base of corolla tube; filaments of lower pair 1.1–1.3 cm long, adnate at 9–10 mm from the base of corolla tube; anthers reniform, 1–1.5 mm long, basifixed, glabrous; staminode absent. Disc ca. 1.3 mm in height, margin orbicular, glabrous. Pistil 21–26 mm long; ovary ca. 2.5 mm long, ca. 1 mm in diam., glabrescent; style 17–22 mm long, ca. 1 mm in diam., pubescent, longer than upper pair of filaments when mature; stigma bilobed, V-shaped, 1–1.5 mm long, glabrous. Capsules linear-oblong, straight, 5–6(–8) × ca. 0.3 cm, glabrous to glabrescent, loculicidal.

**Figure 2.**
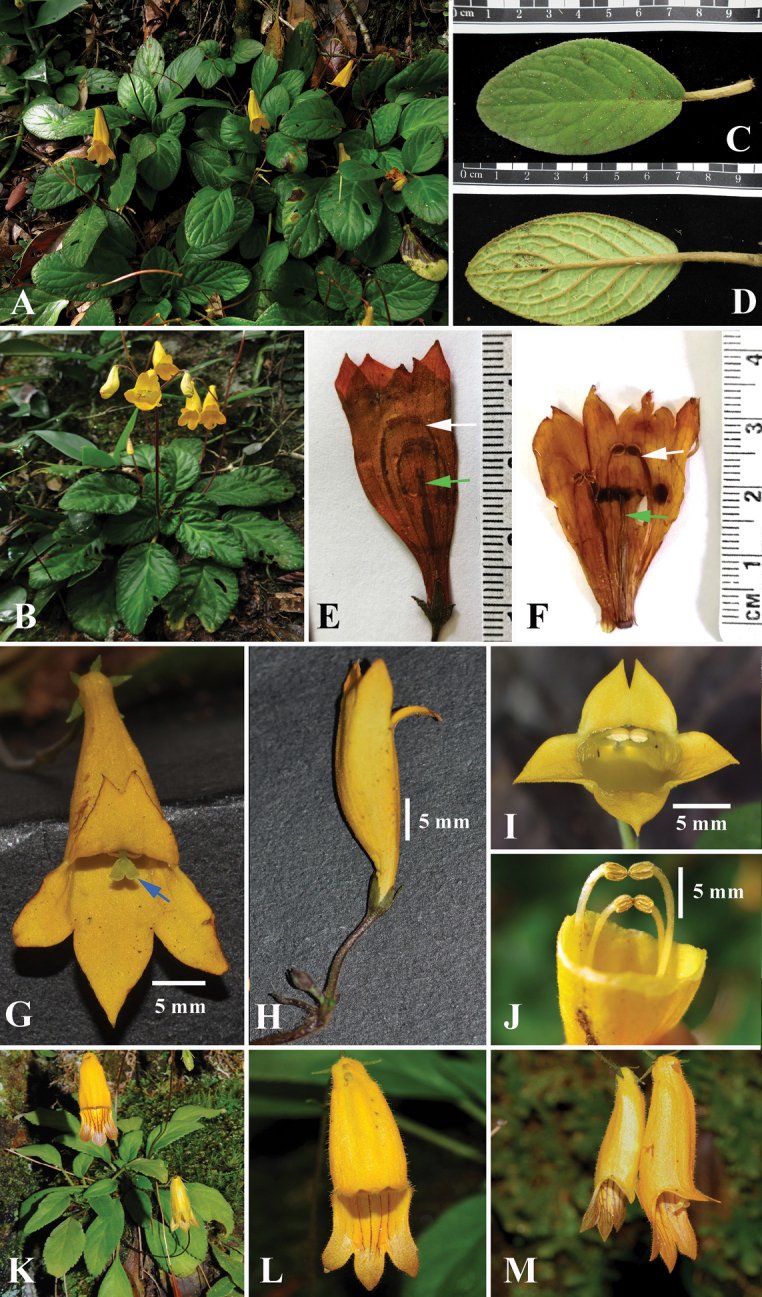
*Oreocharisphuongii* sp. nov. (**A–J**) **A** habitat **B** habit showing cymose inflorescence **C** adaxial leaf surface **D** abaxial leaf surface **E–F** structure of floral parts (two pairs of anther and filament indicated by a white arrow; shape of pistil indicated by a green arrow) **G** frontal view of opened flower showing backwards incurved upper lobes, glabrous inner surface of three lower lobes and a bilobed matured stigma longer than stamens (indicated by a blue arrow) **H** lateral view of opened flower **I** close-up of opened flower (in frontal view) **J** close-up of 2-paired stamens; *O.longifolia* (K–M) **K** habit **L** frontal view of opened flower showing brown to brownish-yellow inner surface of three lower lobes with dark-purple striations **M** lateral view of an opened flower. **A–J** photos by Do Van Truong **K–M** photos by Nicholas Turland.

#### Etymology.

The specific epithet honours Prof. Dr. Vu Xuan Phuong who has contributed significantly to our understanding of Gesneriaceae in Vietnam.

#### Phenology.

Flowering was observed from October to November. Fruiting may occur from November to December.

#### Distribution and habitat.

**Figure 3.**
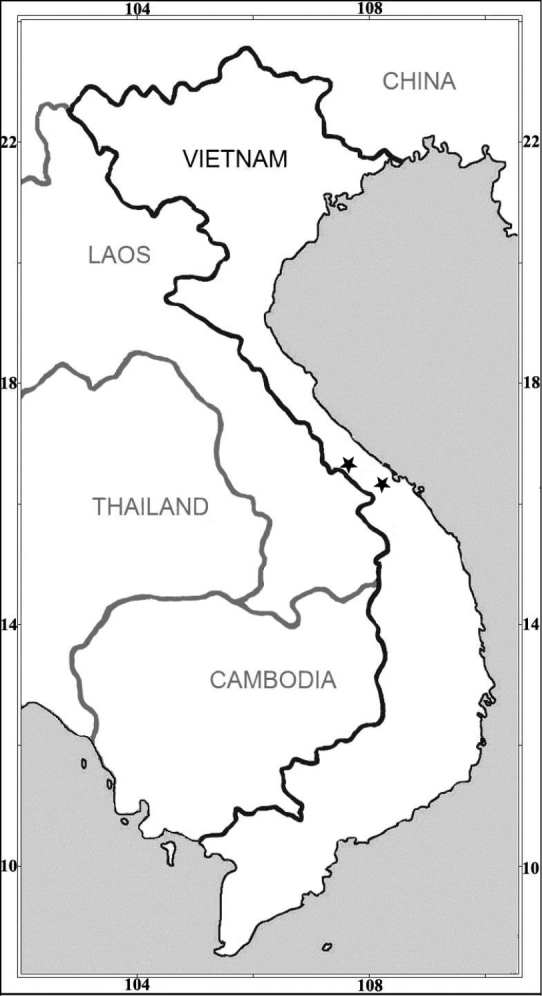
Distribution of *Oreocharisphuongii* sp. nov. from central Vietnam (shown by black stars).

The new species is currently known from some protected forest areas (viz. Dakrong Nature Reserve, Quang Tri Province and Bach Ma National Park, Thua Thien Hue Province) within the Annamite Range, central Vietnam (Fig. 3). The new species grows on moist shady cliffs on the humus-rich limestone hills and moist rocks under evergreen broad-leaved forests, at elevations of 360–650 m.

#### Proposed IUCN conservation status.

Two large-sized populations of *Oreocharisphuongii* were found in the core-zones of Dakrong Nature Reserve, Quang Tri Province and Bach Ma National Park, Thua Thien Hue Province, central Vietnam, which are almost entirely covered by primary forest and are well protected. Furthermore, our field observations of these populations indicated that there are many healthy individuals and seedlings that regenerate in well-protected habitats and there is no immediate threat to the populations from human activities. Thus, the new species is probably not at risk in the near future. This species is preliminarily assessed as Least Concern (LC) according to the IUCN Categories and Criteria (IUCN Standards and Petitions Subcommittee 2019).

#### Notes.

In the size and shape of the corolla and the structure of inflorescences, *O.phuongii* is similar to some species with the acaulescent and rosette-forming stems of the formerly circumscribed *Briggsia*, which previously comprised ca. 30 species and four varieties and was mainly distributed in Bhutan, China, India, Myanmar and Vietnam (Wang et al. 1990, 1998; Vu 2018). Nineteen species and four varieties of acaulescent, rosette forming *Briggsia* (Craib 1920; Pan 1988) were moved to *Oreocharis* s.l. in a later revision (Möller et al. 2011, 2014). Of which, *O.phuongii* is most similar to *O.longifolia* (Craib) Mich.Möller & A.Weber in having peduncles up to 22 cm long, bracts 2, zygomorphic, yellow flowers with tubular corolla, stamen 4 with two pairs of coherent anthers and capsules up to 6 cm long, but it clearly differs from the latter in the shape of leaf blade, leaf base, leaf apex, leaf margin, number of flowers, shape and size of calyx lobes, inner surface of corolla tube and inner surface of three lower corolla lobes. Detailed morphological comparisons of the new species with *O.longifolia* are shown in Table 1 and Figure 2.

**Table 1.** Detailed morphological comparisons of *Oreocharisphuongii* with *O.longifolia*.

Characters	*O.phuongii* sp.nov.	O.longifolia*
Stems	with conspicuous stolons	without stolons
Leaves		
lamina	elliptic to ovate	narrowly elliptic to oblanceolate
base	cuneate to nearly rounded	attenuate
apex	obtuse to almost rounded	acute
margin	crenulate	serrulate
Cymes	1–3-flowered	1–10-flowered
Peduncle length (cm)	12–19(–22)	5.5–22
Calyx		
shape	equally 5-lobed, lobes triangular to lanceolate	subequally 5-lobed, lobes ovate
size (mm)	3–4.5 × 1.5–1.8	5–7 × 2–3
Corolla		
tube size (cm)	2.8–3 × 1.2–1.3	(1–)1.8–2.3 × 0.8–1.6
outer surface of tube	sparsely pubescent	densely trichomes
inner surface of tube	glabrous without striations	sparsely glandular puberulent with purple striations
inner surface of three lower lobes	exclusively yellow without dots and striations	brown to brownish-yellow with purple dots and striations
Distribution	Central Vietnam	South-western China and northern Myanmar

* Morphological characters following Wang et al. (1998) and our own observations.

This new species is the first record of the genus *Oreocharis* occurring in central Vietnam, which raised the species number of *Oreocharis* in Vietnam to nine. Amongst the nine known *Oreocharis* species from Vietnam, the new species shares the yellow to orange corolla with five other species: *O.aurea*, *O.argyrophylla*, *O.grandiflora*, *O.longituba* and *O.tribracteata* (Chen et al. 2017, 2018; Möller et al. 2018). However, it is clearly different from these five species by having a tubular corolla tube (vs. funnel to narrowly funnel corolla tube in *O.argyrophylla*, *O.grandiflora*, *O.longituba*, *O.tribracteata* and urceolate corolla tube in *O.aurea*). Jin et al. (2021) showed that *Oreocharis* sl. could be separated into two clades: Clade A was mainly distributed in SW China and predominantly showed yellow to orange corollas; Clade B was mainly distributed in S and SE China and predominantly showed purple corollas, of which, Clade A includes ca. 20 species. In order to facilitate identification, a key to five yellow to orange species of *Oreocharis* in Vietnam is provided.

#### Additional specimen examined.

Vietnam. Quang Tri Province, Dakrong District, Dakrong Nature Reserve, on moist shady cliffs on the humus-rich limestone hills, 16°29'50.97"N, 107°00'09.25"E, 650 m alt., 18 Oct 2019, Do Van Truong ĐVT 362 (VNMN).

### ﻿A key to yellow to orange species of *Oreocharis* in Vietnam

**Table:** 

1	Corolla tube urceolate, corolla less than 2.5 cm long; anthers free	***Oreocharisaurea***
–	Corolla tube funnel-shaped to tubular, corolla more than 2.5 cm long; anthers coherent in pairs	**2**
2	Corolla tube tubular, abruptly constricted near base	***Oreocharisphuongii***
–	Corolla tube funnel-shaped to narrowly funnel-shaped	**3**
3	Calyx lobes divided to about 2/3 of their length; bracts 3	***Oreocharistribracteata***
–	Calyx lobes free to base or almost so; bracts 2	**4**
4	Leaf base cordate	***Oreocharislongituba***
–	Leaf base cuneate to nearly rounded	**5**
5	Petiole less than 3 cm long; leaf margin crenate; corolla 3.3–3.6 cm long, deep orange	***Oreocharisgrandiflora***
–	Petiole 4–9 cm long; leaf margin serrulate towards apex; corolla 2.5–3.1 cm long, yellow	***Oreocharisargyrophylla***

## Supplementary Material

XML Treatment for Oreocharis phuongii
